# Cationic Nanogels Enable Gold Nanoparticle Immobilization and Regulated Catalytic Activity

**DOI:** 10.3390/polym15081935

**Published:** 2023-04-19

**Authors:** Xin Wang, Xuhong Guo, Martien A. Cohen Stuart, Junyou Wang, Peng Ding

**Affiliations:** State-Key Laboratory of Chemical Engineering, Shanghai Key Laboratory of Multiphase Materials Chemical Engineering, East China University of Science and Technology, Shanghai 200237, China; 18800352773@163.com (X.W.); guoxuhong@ecust.edu.cn (X.G.); martien.cohenstuart@gmail.com (M.A.C.S.); junyouwang@ecust.edu.cn (J.W.)

**Keywords:** nanogel, polyelectrolyte, gold nanoparticles

## Abstract

Polyelectrolyte nanogel consisting of charged network is a prospective platform for developing nanoreactor due to their integrated features of both polyelectrolyte and hydrogel. In this work, cationic poly (methacrylatoethyl trimethyl ammonium chloride) (PMETAC) nanogels with regulated size (30–82 nm) and crosslinking degree (10–50%), has been synthesized by Electrostatic Assembly Directed Polymerization (EADP) method and applied to load gold nanoparticles (AuNPs). Based on the typical reduction reaction of 4-nitrophenol (4-NP), the catalytic performance of the constructed nanoreactor was examined by studying their kinetic process, where the loaded AuNPs exhibited dependent activity on crosslinking degree of nanogels, while independent catalytic activity on nanogel size. Our results validate that, polyelectrolyte nanogels are capable of loading metal NPs and regulating their catalytic performance, therefore demonstrates potential for developing functional nanoreactors.

## 1. Introduction

Metal nanoparticles (NPs) with high catalytic performance and accurate selectivity have gained considerable attention as a kind of efficient catalyst due to their high surface-to-volume ratio and surface energy compared to their bulk materials [1,2,3]. Among the diverse metal NPs, gold nanoparticle (AuNPs) is of particular interests with unique optical properties, large Stokes shift, high photo-stability as well as catalytic properties which have been widely used for imaging, biosensing and catalysis [4,5,6]. For example, gold nanoparticles with ultrasmall size (diameter < 3.0 nm) were prepared by using a cationic chitosan as the template. The designed AuNPs processed significantly enhanced cellular interaction capability and sensitive luminescence response, which showed promising applications in optical intracellular tracking [7]. However, free AuNPs tend to aggregate during the preparation and catalytic process, which therefore severely diminishes their catalytic activity and restricts practical applications [8]. To address this limitation, a suitable support is needed for particles dispersion and prevent their aggregation. Therefore, a variety of nanoscale materials including branched dendrimers [9], polymer brushes [10], silicon dioxide [11], and metal-organic framework (MOF) [12], featured with well-designed functionalities have been developed. For instance, AuNPs was successfully loaded into a multifunctional microsphere with poly (2-dimethylaminoethyl methacrylate) (PDMAEMA) polymer brushes by the in-situ reduction of AuCl_4_^−^ with sodium borohydride. The obtained AuNPs displayed a very stable catalytic activity which still remained 97% of the original activity even after recycling for six times due to the efficient stabilization of the PDMAEMA brushes [13].

Polyelectrolyte nanogels which contain three-dimensional charged polymer network integrate the advanced features of both polyelectrolyte and nanogel, such as high loading capacity, good mechanical properties, and high stability, thus it is generally used as nanocarrier to encapsulate charged cargos for catalytic applications [14,15,16]. On the one hand, their abundant charges are attractive for loading oppositely charged cargos [17,18,19]. For example, cationic nanogels can selectively load negatively charged dyes and release them in a controlled way depending on pH and salt concentration [19]. On the other hand, the charged polymer network in nanogel creates a soft pocket for encapsulating catalytic molecules or nanoparticles, as well as an open environment for reactants diffusion arising from the regulated mesh size [16,20]. The latter endows polyelectrolyte nanogels promising perspective for developing functional nanoreactors. Previous studies have found that, polyelectrolyte nanogels can be applied to load enzymes efficiently without disturbing their (secondary) structures. Moreover, the permeable polymer matrix is profitable for catalytic performance of the loaded enzymes [20]. These inspired results demonstrate generous extensibility of polyelectrolyte nanogels for loading diverse catalytic cargoes, e.g., active metal NPs for developing novel nanoreactors. Though a lot of recent works have focused on the metal NPs preparation and extended their applications, exploiting their structure-activity relationship have not been rarely investigated.

**Scheme 1:**
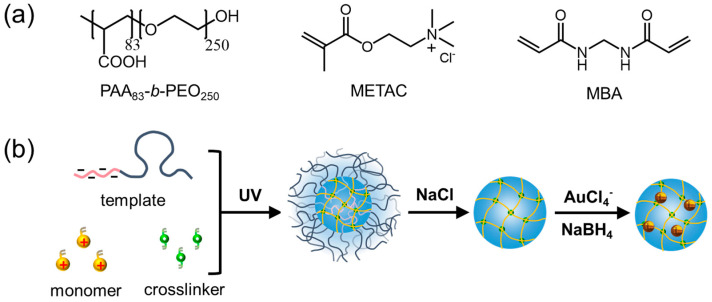
(**a**) Chemical structures of PAA-*b*-PEO template, METAC monomer and MBA cross-linker for preparing PMETAC nanogels used in this study. (**b**) Scheme of the nanogel preparation and gold nanoparticles (AuNPs) immobilization.

In order to address this limitation, in this study we prepared a type of cationic poly (methacrylatoethyl trimethyl ammonium chloride) (PMETAC) nanogels with well controlled particle size and tunable crosslinking degree on the basis of our group recently developed strategy (Electrostatic Assembly Directed Polymerization, EADP) (Scheme 1) [21]. The designed nanogels were further utilized to load AuCl_4_^−^ through electrostatic interaction, and the subsequent in situ reduction created AuNPs embedded inside the nanogels. The nanogel and AuNPs formation were fully investigated by dynamic light scattering (DLS), Ultraviolet-visible (UV-vis) spectroscopy. The morphology of the nanogel and AuNPs loaded nanogel was observed by transmission electron microscope (TEM). The AuNPs loaded nanogel can be used as an effective nanoreactor and their catalytic activity was evaluated by the typical reduction reaction of 4-nitrophenol (4-NP) to 4-aminophenol (4-AP). Furthermore, different impacts of the nanogel particle size and crosslinking degree on the catalytic activity of AuNPs had been fully explored which elaborates the structure-activity relationship between nanogel and active AuNPs.

## 2. Materials and Methods

### 2.1. Reagents

The photoinitiator 2-hydroxy-2-methylpropiophenone (HMP, 96%) and cationic monomer methacrylatoethyl trimethyl ammonium chloride (METAC, 80 wt. % in H_2_O) were bought from TCI (Shanghai, China) Co., Ltd., which were used as received. Diblock polymer poly(acrylic acid-b-ethylene oxide) (PAA_83_-*b*-PEO_250_, polydispersity index 1.09; PAA_44_-*b*-PEO_204_, polydispersity index 1.20) were bought from *Polymer Source Inc.* (Montreal, QC, Canada) which was used as the template. Cationic monomer 2-aminoethyl methacrylate hydrochloride (AEMH) and crosslinker *N*,*N*′-methylenebisacrylamide (MBA, 99%) were purchased from Sigma Aldrich (Shanghai, China). Deuterium oxide (D_2_O, 99.9 atom % D) was obtained from Adamas (Shanghai, China) and used as the deuterated solvent for ^1^H NMR test. 3-Trimethylsilyl propionic acid-d4 sodium salt (TSP, 98 atom % D, with 9H) was purchased from Sigma Aldrich (Shanghai, China) which was used as the internal standard for ^1^H NMR measurement. Chloroauric acid (HAuCl_4_, ≥ 99.9% trace metals basis) was purchased from J&K Chemical Technology (Langfang, China). Sodium borohydride (NaBH_4_, 95%) was purchased from Acros (Pittsburgh, PA, USA). Phosphotungstic acid (H_3_[P(W_3_O_10_)_4_]·xH_2_O) was obtained from Sinopharm Chemical Reagent Co., Ltd. (Shanghai, China). 4-Nitrophenol was brought from J&K Chemical Technology (Langfang, China) and used as the catalytic substrate for AuNPs. Other materials in this study were of commercially analytical grade and were used as received without further purification. Ultrapure water was produced with a Milli-Q water purification system (Millipore, Mingche-D 24UV, Burlington, MA, USA). Carbon coated copper grids were bought from Beijing Zhongjingkeyi Technology Co., Ltd. (Beijing, China).

### 2.2. Characterizations

#### 2.2.1. UV-Vis Spectroscopy

A SHIMADZU 1800 spectrophotometer (Kyoto, Japan) was used to record the UV spectra. The nanogel, Au@nanogel and reaction solutions were put in 1.0 cm quartz cell and recorded at room temperature (25 °C). For testing the catalytic performance of Au@PMETAC nanogel, the UV spectra was monitored every 1 min at 400 nm.

#### 2.2.2. Transmission Electron Microscope (TEM)

TEM observations were carried out using a JEM-1400 electron microscope (JEOL, Tokyo, Japan) instrument operating at 100 kV. The nanogel and Au@nanogel solution were firstly diluted 50-fold times with water. Then a drop (about 10 μL) of the sample solution was placed on a copper grid for 10 min, after that the excess solution was blotted by filter paper. Next, a drop of phosphotungstic acid solution (2.0 wt%, pH 6.0) was soaked onto the surface of the sample-loaded grid for about 15 s to stain the samples, followed by blotting the excess stain and drying at room temperature.

#### 2.2.3. Light Scattering

The size and scattering intensity of the sample were measured with an ALV light scattering (Hessen, Germany) device equipped with a 21 mW argon ion laser with a working wavelength of 632.8 nm. All the sample solution were measured directly without filtration. The scattering angle was fixed at 90°. All samples were tested at room temperature (25 °C). The mean apparent hydrodynamic radius (*R_h_*) was analyzed using the CUMULANT method [22],

$$R_{h}=kTq^{2}/6\pi \eta \Gamma$$

where *k* represents the Boltzmann constant, *T* represents the absolute temperature, *q* represents the scattering vector, *η* represents the viscosity of the solvent, Γ represents the measured average decay rate of the correlation function. The size distribution of nanogel particles hydrodynamic radius was analyzed by the CONTIN method [23,24].

#### 2.2.4. H Nuclear Magnetic Resonance (^1^H NMR)

A BRUKER AVANCE 400 spectrometer operating at 400 MHz was used to record the ^1^H NMR spectra. D_2_O was used as the deuterated solvent to dissolve all the samples after adding sodium chloride to a final salt concentration at 2 M. The spectra were scanned at 25 °C for 32 times. TSP was applied as an internal reference which had a chemical shift at 0 ppm. On the basis of which, the peak at 5.85–5.75 ppm was assigned to the carbon-carbon (C=C) double bond of METAC monomer, the peaks at 3.5–3.0 ppm were assigned to PMETAC polymers [these protons were from −N(CH_3_)_3_ groups]. Since the amount of TSP is already known, the monomer (METAC) and polymer amount (concentration) can be quantitatively analyzed by the integral ratio of monomer or polymer peaks over TSP peak. Monomer conversion can be obtained by analyzing the remained monomer after polymerization with TSP. Most of the converted monomers were crosslinked in the nanogel particles, while some of them were uncrosslinked which escaped from the nanogel during the ultrafiltration purification process. The escaped polymers can be analysed with TSP, from which the final fraction of monomers incorporated in the nanogels can be thereby calculated, which is defined as the final particle yield.

### 2.3. Preparation of PMETAC Nanogels

PMETAC nanogels were prepared according to the previously reported method [19,21]. The charge mixing ratio was fixed at a stoichiometric ratio of 1:1, and the concentration is set as 20 mM. Typically, anionic template PAA-*b*-PEO (8.2 mg), cationic METAC monomer (8.3 mg, 0.04 mmol), crosslinker MBA (0.62 mg, 0.004 mmol), and photoinitiator HMP (0.131 mg, 8 × 10^−4^ mmol) were mixed into water to a final volume of 2 mL. The pH of the mixed reaction solution was adjusted to pH 8.5 using 2.0 M sodium hydroxide. The solution was put in a 10 mL Schlenk tube which was sealed and de-oxygenated by bubbling with nitrogen for 30 min. After that the tube was placed under the UV light and exposed for 3 h (Note that the UV light was cooled by a cooling water recirculation system to avoid the temperature increasing). The reaction was then stopped by directly opening the tube and exposing it to air. Referring to preparing the nanogel with different sizes, the above-mentioned recipe and reaction conditions were used while tuning the salt concentration of solution at 0 mM, 5 mM, 10 mM, 20 mM, 30 mM. Nanogels synthesized at different pH were carried out according to the above-mentioned recipe and reaction conditions but using different pH (4.5, 5.5, 6.5, 7.5 and 8.5). In order to separate template, salt concentration of the reaction solution was tuned to 2 M, followed by centrifuging with an Amicon Ultra-4 centrifugal filter (molecular weight cut off = 100 kDa) using 2 M NaCl as eluent for six times. Finally, the nanogel was obtained by dialyzing against pure water to remove the salt and used for further study.

### 2.4. Synthesis of Au@PMETAC Nanogels

Au@PMETAC nanogels were prepared according to the previously reported method [9]. Typically, the pH of 1 mL obtained nanogel solution was adjusted to 3.0 using 1 M hydrogen chloride. Then 100 μL of HAuCl_4_ (10 mM) were added to the above solution. After stirring for 20 min, 200 μL sodium borohydride (NaBH_4_, 200 mM) was added to the resulting solution. The color of solution changed very quickly from colorless to a dark brown in few seconds after the addition of NaBH_4_, indicating the reduction of Au^3+^ to Au. Finally, Au@PMETAC nanogels were purified by dialysis against pure water to remove the free metal precursors, unreacted sodium borohydride and generated salt.

### 2.5. Catalytic Performance of Au@PMETAC Nanogel

The catalytic performance of Au@PMETAC nanogel was evaluated by the typical reduction reaction of 4-nitrophenol (4-NP) to 4-aminophenol (4-AP). Typically, 1 mL solution containing 10 μL of aqueous 4-nitrophenol (10 mM) and 100 μL NaBH_4_ (100 mM) was prepared at room temperature. The reaction started by adding 2.5 μL of Au@PMETAC nanogel into the above mixture. Then the time-dependent UV absorbances were determined by UV-vis spectrophotometer to measure the concentration change of 4-nitrophenol (about 400 nm).

## 3. Results

### 3.1. Preparation of PMETAC Nanogels

Electrostatic Assembly Directed Polymerization (EADP) method was used to prepare PMETAC nanogels [19,21]. Specifically, with a polyion-neutral di-block copolymer as the template, an oppositely charged monomer and crosslinker are in-situ polymerized together. As illustrated in Scheme 1, poly (acrylic acid-*b*-ethylene oxide) (PAA_83_-*b*-PEO_250_) is selected as the anionic template polymer, methacrylatoethyl trimethyl ammonium chloride (METAC) is selected as the cationic monomer, and *N*,*N*′-methylenebis (acrylamide) (MBA) is selected as the cross-linker. Upon reaction, cationic METAC monomers and MBA crosslinkers polymerize and grow together which simultaneously assembly with the anionic PAA-*b*-PEO template to form polyion complex (PIC) micelles. Subsequently, the formed PIC micelles were dissociated by 2 M NaCl [25,26]. PMETAC nanogels were separated from the template by centrifugation with an appropriate semipermeable membrane (molecular weight cut off =100 kDa), followed by dialyzing against pure water to remove the salt.

**Figure 1:**
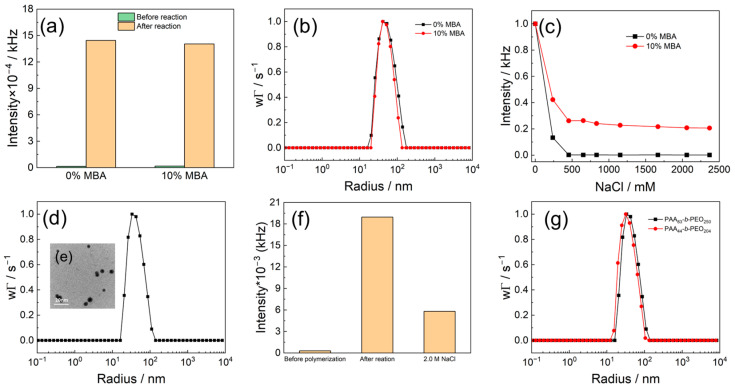
(**a**) Light scattering intensity of reaction solution prepared with and without 10% MBA cross-linkers in the presence of PAA_83_-*b*-PEO_250_ template before and after UV light irradiation for 3 h. (**b**) Size distribution of reaction solution prepared with and without 10% MBA cross-linkers in the presence of PAA_83_-*b*-PEO_250_ template after UV light irradiation for 3 h. (**c**) Light scattering intensity of formed polyion complex micelles prepared with PAA_83_-*b*-PEO_250_ template titrated with sodium chloride. Size distribution (**d**) and TEM image (**e**) of PMETAC nanogel after removing PAA_83_-*b*-PEO_250_ template. (**f**) Light scattering intensity of mixture before and after polymerization and the reaction mixture in 2.0 M NaCl, with PAA_44_-*b*-PEO_204_ as the template. (**g**) Size distribution of PMETAC nanogel prepared with different template.

The successful formation of the nanogel is characterized by light scattering and salt titration. First, micelles with and without the MBA crosslinker were prepared in the presence of PAA-*b*-PEO di-block copolymer template. As demonstrated in Figure 1a, the light scattering intensity of the reaction mixture increases significantly after the UV light irradiation, suggesting the successful polymerization and assembly between the formed PMETAC polymer and PAA-*b*-PEO template. Both the reaction solution (with and without the MBA crosslinker) have very similar hydrodynamic radii with narrow size distribution, which indicates the MBA crosslinker shows negligible impact on the polymerization and self-assembly process (Figure 1b). However, crosslinker indeed influences the salt response behavior of the micelles. For micelles prepared without cross-linker, the light scattering intensity decreases with increasing salt concentration; the particles dissociate completely around 500 mM, which is a typical salt response behavior of polyion complex micelles (Figure 1c) [26]. For micelles prepared with 10% cross-linker, the scattering intensity first decreases with the increasing salt concentration, then it reaches to a final plateau of about 20% of the original intensity. The above results verify that the introduction of MBA crosslinker barely influence the polymerization and self-assembly process, while it indeed successfully crosslinks the PMETAC polymer into a nanogel network. After removing the PAA-*b*-PEO template through ultra-centrifugation, PMETAC nanogel can be obtained with a narrow size distribution and spherical morphology (Figure 1d,e). The quantitative analysis of the sample with ^1^H NMR using TSP as internal standard reveals that more than 90% METAC monomer and almost 100% crosslinker converted into polymer network, and the final particle yield is about 60%. We also varied the diblock polymer (PAA_44_-*b*-PEO_204_) and studied its influence on nanogels formation. The results showed that PMETAC nanogel with a hydrodynamic radius of about 31 nm and a narrow size distribution can be prepared with the PAA_44_-*b*-PEO_204_ as the template, which indicated that template had little negligible effect on the nanogel formation.

**Figure 2:**
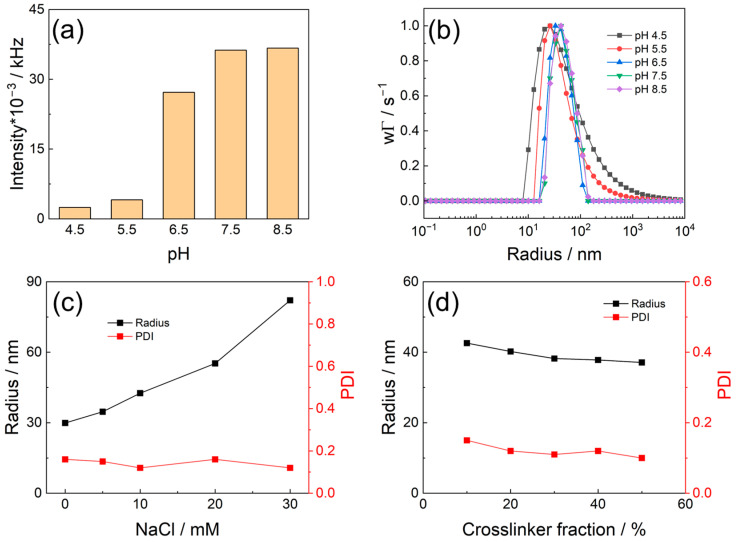
(**a**) Light scattering intensity of particles after UV light irradiation for 3 h prepared at different pH. (**b**) Size distribution of PMETAC nanogel prepared at different pH after removing template. Hydrodynamic radius and polymer dispersity index (PDI) of PMETAC nanogel prepared at different added sodium chloride concentration (**c**) and crosslinker fraction (**d**). Nanogel synthesis was carried out at charge mixing ratio 1:1, cross-linker fraction 10% and with different pH or sodium chloride concentration.

Since the ionization degree of PAA-*b*-PEO template is pH dependent [27], we first investigate and optimize the pH on the synthesis of PMETAC nanogels. The formation and particle size of the nanogels were characterized by light scattering. As shown in Figure 2a,b, particles formed at pH 4.5 show relatively low light scattering intensity and broad size distribution. Apparently, the protonated carbonyl group (−COOH) of PAA-*b*-PEO template at low pH leads to the insufficient electrostatic interaction between the building polymers and the template, which results in the poor control of the PMETAC nanogel. The light scattering intensity increases, along with an improved size distribution when pH increases. In the pH range of 6.5 to 8.5, well controlled PMETAC nanogels with hydrodynamic radius of 30 nm can be obtained. Clearly, sufficient charge interaction is essential to create a confined environment for better control on the polymerization, self-assembly and nanogel formation. The preferred pH is 8.5 where the −COOH groups of the PAA-*b*-PEO template are fully protonated, thus leads to the highest scattering intensity as well as narrow size distribution.

As the electrostatic interaction relies on ionic strength [25,26], the size of PMETAC nanogels can be regulated by adding different amount of sodium chloride in the reaction solution during the polymerization. As displayed in Figure 2c, the obtained PMETAC nanogels display increased hydrodynamic radius from 30 nm to 82 nm with the increasing added sodium chloride from 0 to 30 mM while keeping their narrow size distribution. Apparently, the electrostatic interaction between the forming charged building blocks and the template is weaken by the added sodium chloride, leading to an enhanced mobility which is favorable for creating nanogels with larger particle size [28,29]. The crosslinking degree of PMETAC nanogel is controlled by synthesizing nanogels with different input crosslinker fractions of 10%, 20%, 30%, 40% and 50% (with respect to molar concentration of METAC monomer). Figure 1d indicates that the hydrodynamic radius of PMETAC nanogels decrease slightly with the increasing crosslinker fractions, while the size distribution remains narrow with a PDI value below 0.2. Obviously, the higher crosslinker fraction creates the compact network inside the nanogel which decreases nanogels’ size. Results so far confirm the successful preparation as well as the regulated size (30–82 nm) and crosslinking degree (10–50%) of PMETAC nanogels, which provides diverse platforms for loading AuNPs and investigating their catalytic performance.

### 3.2. Synthesis of Au@PMETAC Nanogels

**Figure 3:**
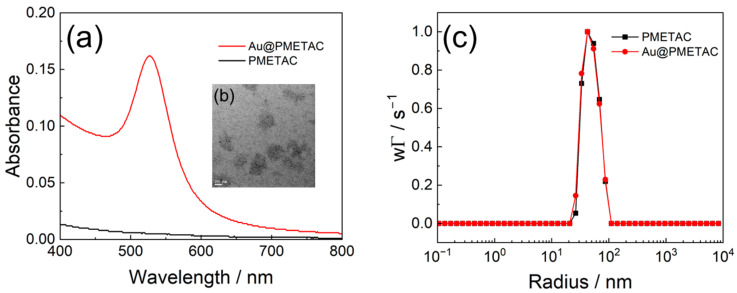
(**a**) UV-Vis spectra of PMETAC and Au@PMETAC nanogels. (**b**) TEM image of Au@PMETAC nanogel. (**c**) Size distribution of PMETAC and Au@PMETAC nanogels. Nanogel synthesis was carried out at pH 8.5, charge mixing ratio 1:1, cross-linker fraction 10% and 0 mM NaCl.

AuNPs were embedded inside the PMETAC nanogels through in-situ growth method [8], that is, anionic AuCl_4_^−^ ions were first absorbed inside the cationic PMETAC nanogel through charge interaction followed by reduction reaction (triggered by NaBH_4_). The typical absorbance band around 530 nm (Figure 3a) along with the TEM image (Figure 3b) confirm the successful loading of AuNPs [30,31]. The light scattering suggests that the size of PMETAC nanogels hardly change after loading AuNPs (Figure 3c). The space between the nanogels contains hardly free AuNPs, which indicates PMETAC nanogels can be an ideal platform for untaken of AuCl_4_^−^ ions and encapsulation of AuNPs.

### 3.3. Catalytic Performance of Au@PMETAC Nanogel

**Figure 4:**
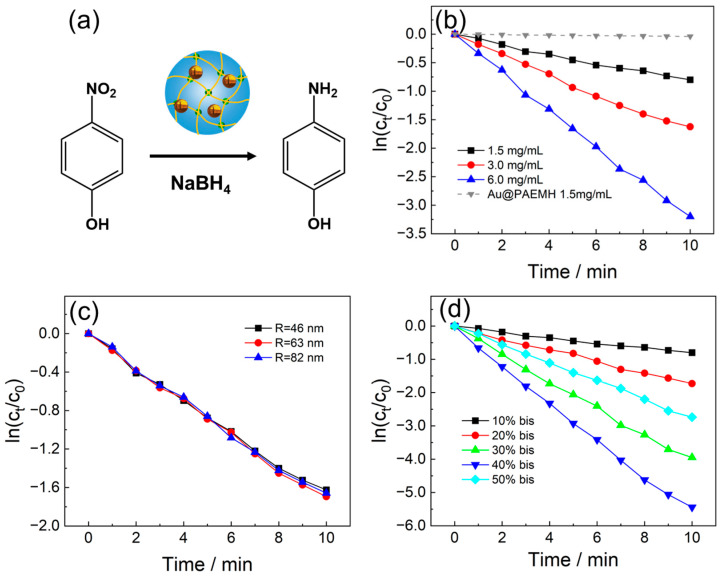
(**a**) Reduction of 4-nitrophenol (4-NP) to 4-aminophenol catalyzed by Au@PMETAC nanogel. (**b**) Comparison of ln(*c_t/_c*_0_) vs. time for reduction of 4-NP catalyzed by Au@PMETAC at different nanogel concentrations and Au@PAEMH nanogel. Comparison of ln(*c_t/_c*_0_) vs. time for reduction of 4-NP catalyzed by Au@PMETAC with PMETAC nanogel prepared at different sizes (**c**) and crosslinking degrees (**d**).

The catalytic performance of the Au@PMETAC nanogels with different nanogel structure was evaluated by a typical reduction reaction of 4-nitrophenol (4-NP) to 4-aminophenol (4-AP) by sodium borohydride (NaBH_4_) (Figure 4a). Substrate 4-NP shows a typical UV absorption peak around 400 nm which decreased gradually when AuNPs@PMETAC nanogel was added to catalyze the reduction reaction [32]. The reaction kinetic process was investigated in this study to evaluate the catalytic activity of Au@PMETAC nanogel. In order to do that, the concentration of NaBH_4_ was set much higher than that of 4-NP, so the kinetics of this reaction can be treated as a pseudo-first-order reaction with regard to the 4-NP, during which the concentration of NaBH_4_ can be assumed as constant [33]. For all the experiments, the initial reaction concentrations of 4-NP and NaBH_4_ were set at 0.1 mmol/L and 10 mmol/L, respectively. The concentration of Au nanoparticles was kept at 0.002 mmol/L. The concentration of NaBH_4_ was 100 times higher than that of 4-NP. Under this reaction condition, the reaction kinetics can be expressed as: −*dc_t/_dt = k_app_c_t_*, where *c_t_* is the concentration of 4-NP at reaction time *t*, *k_app_* is the apparent rate constant. By plotting the concentration ln(*c_t/_c*_0_) of 4-NP versus *t*, the apparent rate constant *k_app_* can be obtained. First, the influence of monomer (METAC with quaternary amine, AEMH with primary amine) type was studied (Figure 4b). It turns out that the catalytic activity of Au@PMETAC was almost 20 times higher than that of Au@PAEMH nanogel, which is due to the PAEMH nanogel network is uncharged and tends to shrink due to the deprotonation of the amino groups under basic environment. Then, the ratio of AuCl_4_^−^ to PMETAC nanogels was studied. As shown in Figure 4b, a linear relationship was obtained between the concentration ln(*c_t/_c*_0_) of 4-NP and reaction time, which is consistent with the first-order kinetics. The *k_app_* increases when the nanogel concentration increased from 1.5 mg/mL to 6.0 mg/mL. It seems that, the increased nanogel concentration leads to a better dispersion of AuNPs and consequently enhanced catalytic activity.

We then studied the effect of nanogel size on AuNPs’ catalytic activity. PMETAC nanogels with hydrodynamic radius at 46 nm, 63 nm and 82 nm loading same amount of AuNPs were selected to evaluate the activity. Interestingly, the nanogels with different particle sizes show nearly same reaction rate constant. We believe that the pore size of nanogel with different radius is bigger enough for reactant and product diffusion, which exhibits limited influence on AuNPs activity. In contrast, the activity of the loaded AuNPs displays a strong dependency on crosslinking degree of PMETAC nanogels. The catalytic activity first increases then decreases with increasing crosslinking degree ranging from 10% to 50%. The highest catalytic activity occurs at 40% crosslinking degree (Figure 4d). We attribute this to the balance of the rigid network that disperses the AuNPs and the pore size of the nanogel that govern the substrate and product diffusion. When the crosslinking degree is below 40%, the rigidity of the network increases with increasing crosslinking degree, which provides a favorable environment for AuNPs dispersion. The substrate and product diffusion are hardly interrupted and therefore leading to increased catalytic activity. This finding may imply that, the pose size of the nanogel under this range of crosslinking degree (10–40%) is larger enough for reactant diffusion. Further increasing crosslinking degree up to 50% leads to decreased pore size to a boundary point that diminishes substrate diffusion and consequently declines the catalytic activity of AuNPs.

## 4. Conclusions

In summary, cationic poly (methacrylatoethyl trimethyl ammonium chloride) (PMETAC) polyelectrolyte nanogels with controlled size (30–82 nm) and tunable crosslinking degree (10–50%) were synthesized by the EADP (Electrostatic Assembly Directed Polymerization) method. The strategy involves polymerization of METAC monomer together with crosslinker in the presence of an anionic-neutral diblock copolymer as the template. Tuning the added salt concentration allows to effectively regulate the nanogel radius ranging from 30 nm to 82 nm without disturbing their narrow size distribution. Moreover, increasing crosslinker fraction during the polymerization obtains the nanogels with slightly decreased size yet with more rigid gel network with smaller pore size. The obtained cationic PMETAC nanogel is an ideal carrier for loading AuNPs which can be used as nanoreactor for reduction of 4-nitrophenol to 4-aminophenol. The catalytic activity (*k_app_*) of the embedded AuNPs can be enhanced with the increasing nanogel concentration due to a better dispersion of AuNPs. The nanogel size has limited influence on the catalytic activity, because the pore size of nanogel with different radius is bigger enough for reactant and product diffusion. While the crosslinking degree of the nanogel exhibits a significant impact on the activity. It first rises then declines with increasing crosslinking degree, and the optimal activity was found at 40% crosslinking degree, which can be attributed to the balance of the rigid network that disperses the AuNPs and the pore size of the nanogel that govern the substrate and product diffusion. The designed polyelectrolyte nanogels shall be extended for loading of other catalytic metal nanoparticles, and hence feature great potential as prospective platforms for developing novel nanoreactors.

## Data Availability

All the data of this study are included in the manuscript.

## References

[1] Lara P., Martínez-Prieto L.M. (2021). Metal Nanoparticle Catalysis. Catalysts.

[2] Feng S., Song X., Liu Y., Lin X., Yan L., Liu S., Dong W., Yang X., Jiang Z., Ding Y. (2019). In situ formation of mononuclear complexes by reaction-induced atomic dispersion of supported noble metal nanoparticles. Nat. Commun..

[3] Gao C., Lyu F., Yin Y. (2021). Encapsulated Metal Nanoparticles for Catalysis. Chem. Rev..

[4] Montes-García V., Squillaci M.A., Diez-Castellnou M., Ong Q.K., Stellacci F., Samorì P. (2021). Chemical sensing with Au and Ag nanoparticles. Chem. Soc. Rev..

[5] Masse F., Ouellette M., Lamoureux G., Boisselier E. (2019). Gold nanoparticles in ophthalmology. Med. Res. Rev..

[6] Jia Z., Amar M.B., Yang D., Brinza O., Kanaev A., Duten X., Vega-González A. (2018). Plasma catalysis application of gold nano-particles for acetaldehyde decomposition. Chem. Eng. J..

[7] Zhu J., He K., Dai Z., Gong L., Zhou T., Liang H., Liu J. (2019). Self-Assembly of Luminescent Gold Nanoparticles with Sensitive pH-Stimulated Structure Transformation and Emission Response toward Lysosome Escape and Intracellular Imaging. Anal. Chem..

[8] Mazloomi-Rezvani M., Salami-Kalajahi M., Roghani-Mamaqani H., Pirayesh A. (2017). Effect of surface modification with various thiol compounds on colloidal stability of gold nanoparticles. Appl. Organomet. Chem..

[9] Hove J.B.T., Wang J., van Oosterom M.N., van Leeuwen F.W.B., Velders A.H. (2017). Size-Sorting and Pattern Formation of Nanoparticle-Loaded Micellar Superstructures in Biconcave Thin Films. ACS Nano.

[10] Zhao F., Li L., Tian Y.-C., Liu J.-J., Wang J.-J., Zhou Z.-M., Lv C.-X., Guo X.-H. (2015). Preparation of Au/Ag multilayers via layer-by-layer self-assembly in spherical polyelectrolyte brushes and their catalytic activity. Chin. J. Polym. Sci..

[11] Mota-Santiago P., Kremer F., Rizza G., Dufour C., Khomenkov V., Notthoff C., Hadley A., Kluth P. (2020). Ion shaping of single-layer Au nanoparticles in amorphous silicon dioxide, in silicon nitride, and at their interface. Phys. Rev. Mater..

[12] Yan R., Zhao Y., Yang H., Kang X.-J., Wang C., Wen L.-L., Lu Z.-D. (2018). Ultrasmall Au Nanoparticles Embedded in 2D Mixed-Ligand Metal-Organic Framework Nanosheets Exhibiting Highly Efficient and Size-Selective Catalysis. Adv. Funct. Mater..

[13] Liu B., Zhang D., Wang J., Chen C., Yang X., Li C. (2013). Multilayer Magnetic Composite Particles with Functional Polymer Brushes as Stabilizers for Gold Nanocolloids and Their Recyclable Catalysis. J. Phys. Chem. C.

[14] Yuan F., Wang S., Chen G., Tu K., Jiang H., Wang L.Q. (2014). Novel chitosan-based pH-sensitive and disintegrable polyelectrolyte nanogels. Colloid Surf. B-Biointerfaces.

[15] Zhou L., Gao Y., Cai Y., Zhou J., Ding P., Stuart M.A.C., Wang J. (2022). Controlled synthesis of PEGylated polyelectrolyte nanogels as efficient protein carriers. J. Colloid Interface Sci..

[16] Ni J., Wan Y., Cai Y., Ding P., Stuart M.A.C., Wang J. (2022). Synthesis of Anionic Nanogels for Selective and Efficient Enzyme Encapsulation. Langmuir.

[17] Spencer D.S., Shodeinde A.B., Beckman D.W., Luu B.C., Hodges H.R., Peppas N.A. (2021). Cytocompatibility, membrane disruption, and siRNA delivery using environmentally responsive cationic nanogels. J. Control. Release.

[18] Knipe J.M., Strong L.E., Peppas N.A. (2016). Enzyme- and pH-Responsive Microencapsulated Nanogels for Oral Delivery of siRNA to Induce TNF-alpha Knockdown in the Intestine. Biomacromolecules.

[19] Ding P., Liu W., Guo X., Stuart M.A.C., Wang J. (2021). Optimal synthesis of polyelectrolyte nanogels by electrostatic assembly directed polymerization for dye loading and release. Soft Matter.

[20] Cai Y., Ding P., Ni J., Zhou L., Ahmad A., Guo X., Stuart M.A.C., Wang J. (2021). Regulated Polyelectrolyte Nanogels for Enzyme Encapsulation and Activation. Biomacromolecules.

[21] Ding P., Huang J., Wei C., Liu W., Zhou W., Wang J., Wang M., Guo X., Stuart M.A.C., Wang J. (2020). Efficient and Generic Prep-aration of Diverse Polyelectrolyte Nanogels by Electrostatic Assembly Directed Polymerization. CCS Chem..

[22] Koppel D.E. (1972). Analysis of Macromolecular Polydispersity in Intensity Correlation Spectroscopy: The Method of Cumulants. J. Chem. Phys..

[23] Provencher S.W. (1982). A constrained regularization method for inverting data represented by linear algebraic or integral equations. Comput. Phys. Commun..

[24] Provencher S.W. (1982). Contin: A general purpose constrained regularization program for inverting noisy linear algebraic and integral equations. Comput. Phys. Commun..

[25] Van Der Kooij H.M., Spruijt E., Voets I.K., Fokkink R., Stuart M.A.C., Van Der Gucht J. (2012). On the Stability and Morphology of Complex Coacervate Core Micelles: From Spherical to Wormlike Micelles. Langmuir.

[26] Voets I.K., de Keizer A., Stuart M.A.C. (2009). Complex coacervate core micelles. Adv. Colloid Interface Sci..

[27] Spruijt E., Westphal A.H., Borst J.W., Stuart M.A.C., van der Gucht J. (2010). Binodal Compositions of Polyelectrolyte Complexes. Macromolecules.

[28] Liu Y., Winter H.H., Perry S.L. (2017). Linear viscoelasticity of complex coacervates. Adv. Colloid Interface Sci..

[29] Liu Y., Momani B., Winter H.H., Perry S.L. (2017). Rheological characterization of liquid-to-solid transitions in bulk polyelectrolyte complexes. Soft Matter.

[30] Li Z., Wan J., Zhang Y., Dang C., Pan F., Fu J. (2021). Influences of petroleum hydrocarbon pyrene on the formation, stability and antibacterial activity of natural Au nanoparticles. Sci. Total Environ..

[31] Tu T., Zhou W., Wang M., Guo X., Li L., Stuart M.A.C., Wang J. (2020). One-Pot Synthesis of Small and Uniform Gold Nanoparticles in Water by Flash Nanoprecipitation. Ind. Eng. Chem. Res..

[32] Zhang P., Shao C., Zhang Z., Zhang M., Mu J., Guo Z., Liu Y. (2011). In situ assembly of well-dispersed Ag nanoparticles (AgNPs) on electrospun carbon nanofibers (CNFs) for catalytic reduction of 4-nitrophenol. Nanoscale.

[33] Lu Y., Mei Y., Ballauff M., Drechsler M. (2006). Thermosensitive Core−Shell Particles as Carrier Systems for Metallic Nanoparticles. J. Phys. Chem. B.

